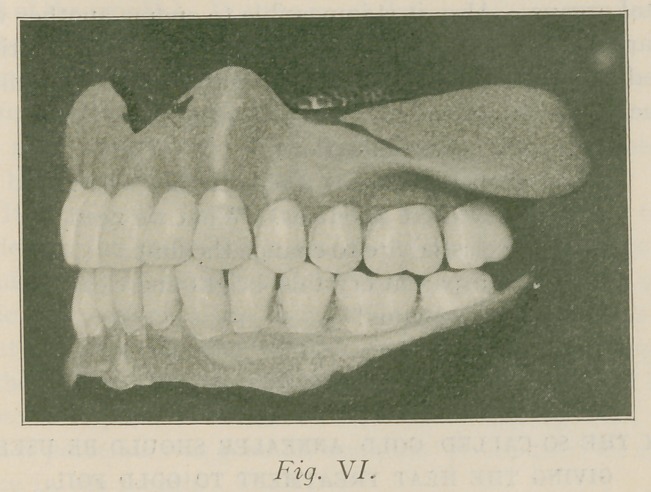# Proving the Articulation, Contour and Expression

**Published:** 1906-08-15

**Authors:** George H. Wilson

**Affiliations:** Cleveland, Ohio


### PROVING THE ARTICULATION, CONTOUR AND EXPRESSION.

BY GEORGE H. WILSON, D.D.S., CLEVELAND, OHIO.

The difficulties of restoring the lost dental organs increases in the ratio to the number of teeth lost. There are some exceptions to this rule, but they are of minor importance and will not be co isidered All small partial cases require little more than mechanical art and the ability to reproduce that which is before our eyes; while the loss of the full upper or lower denture requires greater artistic ability, the loss of both upper and lower teeth commands manipulative ability and the exercise of the esthetic sense to a far greater extent than any other procedure in the field of dental operations.

True are the words of Pope:—

"Whoever thinks a faultless piece to see,
Thinks what ne'er was, nor is, nor e'er shall be."

Surely it is our duty to constantly strive toward perfection. It is with this thought in mind that I desire to offer a few suggestions upon "Proving the articulation, contour and expression" in constructing a full upper and lower vulcanite denture.

That a vulcanite base plate is absolutely necessary for artistic prosthesis in vulcanite is a broad statement, but tenable. By this means the retention of the wax models is as perfect as the finished dentures. With this solid foundation the superstructure of wax can be carved and molded to the mouth.

A practical case will best illustrate the principles we desire to elucidate.

The subject we have chosen is about forty years of age, and for fifteen years been edentulous. She is of a strongly marked sanguine temperament with a nervous temperment modification. The teeth selected were the S. S. W. natural forms, mold number 212, shade 40

Fig. III
Fig II.
Fig. I

Fig. I. Presents a Profile view of the edentulous patient Fig. IV represents the teeth, wax and vulcanite base plates mounted upon the articulator. This illustration also shows the festoon and marginal strings in position, which should not be in this stage of the operation.

*Proving the occlusion.* Having removed the dentures from the articulator, chilled the wax and moistened the maxillary surfaces, they are adjusted in the mouth. The mesio-distal relation of the dentures is studied by repuesting the patient to

*Fig. IV.*

open and close the mouth, also have the patient take a swallow of water, repeat these procedures until satisfied that the patient can not retrude the mandible beyond the position at which the teeth were arranged upon the articulator. Instruct the patient to hold the teeth firmly together, then endeavor to pass a thin blade cement spatula between the molars and biscuids first upon one side and then upon the other side of the mouth. If the occlusion is correct the pressure will be found equal upon the two sides of the mandible. Should the occlusion prove defective it must be corrected; generally this can be done by softening the wax and manipulating the teeth both out of and within the mouth without placing upon the articulator. When they are prope erly occluded they are removed from the mouth, washed and dried with a cloth and with a blast of air (compressed air foot bellows), all moisture is dislodged from between the teeth and wax. The teeth are then thoroughly luted to place by a hot wax spatula and if necessary additional wax added. The other requirements for articulation are not tested until after a satisfactory setting of the teeth has been secured.

*Harmonizing:* It is presumed that teeth of suitable form, size and color to meet the requirements of the case have been used, so that all that remains to be done is to prove the setting of the teeth, both collectively and individually.

*Profile:* Fig. II is the profile view of the patient with the detures in situ. The operator should take his position at the side and a few feet away from the patient. Endeavor to have the teeth in occlusion and the muscles at rest. Notice the perpendicular line of the face, observe whether it belongs to the straight, convex or concave type. The lips and the length of the so-called lower-third of the face must be brought into harmony.

*Full face view:* The operator should take a position a few feet in front of the patient, and while the face is in repose study to see if harmony exists between the fixed points of the face, that is the chin, the molar and the frontal eminences. Attention should now be given to the naso-labio-malar triangles. These triangles were formerly oc--cupied by the apices of the cuspid teeth and must be restored by the cuspid eminence of the artificial denture. Attention is next given to the incisive and cuspid fossae, care must be taken that these places are not too full. Free relief should be given the fraenum labii. The Practice of cutting the labial and buccal strings is most reprehensible.

After the anterior gum portion of the upper denture has been properly contoured the corresponding portions of the lower denture should receive equal attention, although it will not be so marked in outline.

Engage the patient in conversation and laughter, the operator at the time observing the teeth; note that the occlusal plane of the teeth is at right angles to the medium line of the face, and that the incisors and cuspids properly incline toward the median line. See Fig. III. The dentures are now collectively harmonious.

*Individualizing the teeth:* A mirror should be given the patient and her assistance enlisted in individualizing each tooth. It is not necessary to reproduce all the irregularities that existed in the natural tooth, but a modified form of the

*Fig. V.*

irregularity may give a pleasing personality and serve to differentiate between the mouth of the patient and that of Mr. Blank.

The dentures should be removed from the mouth, thoroughly dried and if any of the teeth are loose they must be securely fixed in place. If buccal restorations are required, wax may be added to produce the necessary contour.

*The articulation:* Is proven by having the patient carefully close the teeth in every conceivable position and

*Fig. VI.*

by holding the lips apart, the operator may by the aid of a mouth mirror discein any imperfections of articulation and should at once correct it. The patient is then dismissed.

To secure against accidents in the succeeding procedure the upper cast should be removed from the articulator, place the dentures and casts in position, and refasten the cast to the articulator.

The wax models are now ready for the strings as shown in Fig. IV, also for encasing in number sixty tin foil. Figs. V. and VI show the complete dentures.

The mechanical procedure for constructing the vulcanite denture by the double vulcanization method may be found in the March 1903 number of the Dental Summary. This method is especially adapted to developing the esthetic features of vulcanite work.

While I have attempted to point out the various steps for proving the contour and expression, it not possible to go into all the minor details, for that would require a dissertation upon the temperaments, the muscles of expression, a description of the osseous formation of the oral region and the external changes that take place as the result of the lost dental organs. Also, it is impossible to endow another with the artistic or esthetic sense. This artistic sense is sometimes called genius. Someone has said that "Genius is infinite capacity for painstaking." Longfellow says:

"All the means of action—
The shapeless masses, the materials—
Lie everywhere about us. What we need
Is the celestial fire to change the flint
Into transparent crystals, bright and clear.
That fire is genius!"

## Figures and Tables

**Fig. I. f1:**
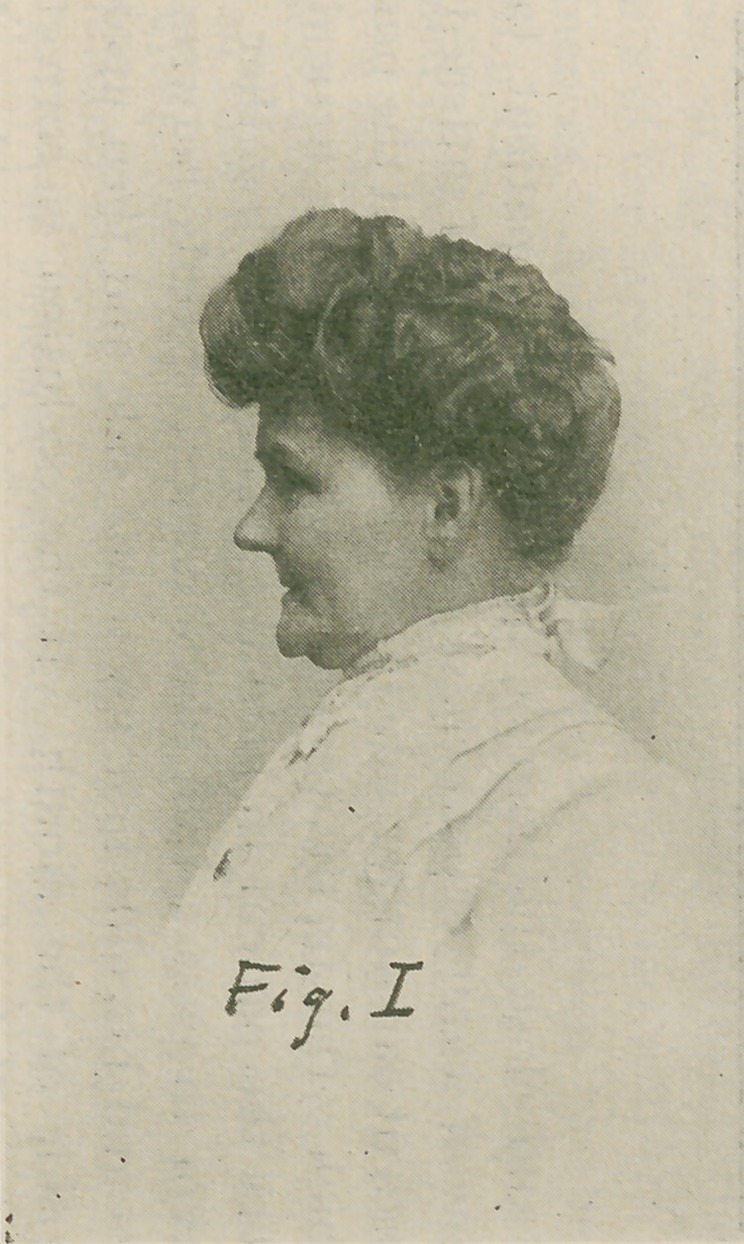


**Fig. II. f2:**
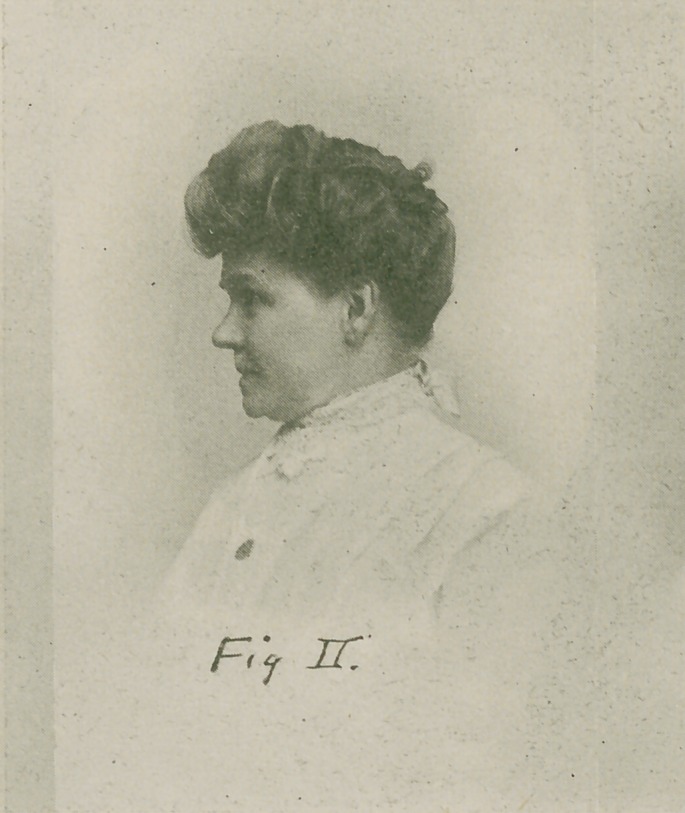


**Fig. III. f3:**
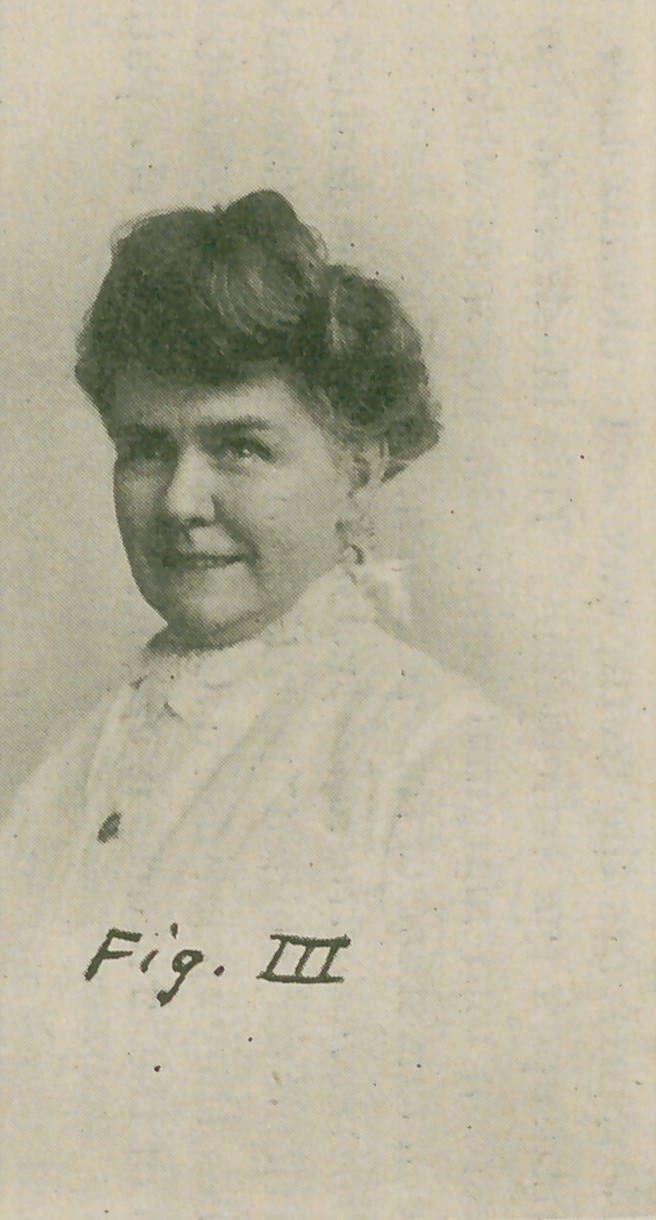


**Fig. IV. f4:**
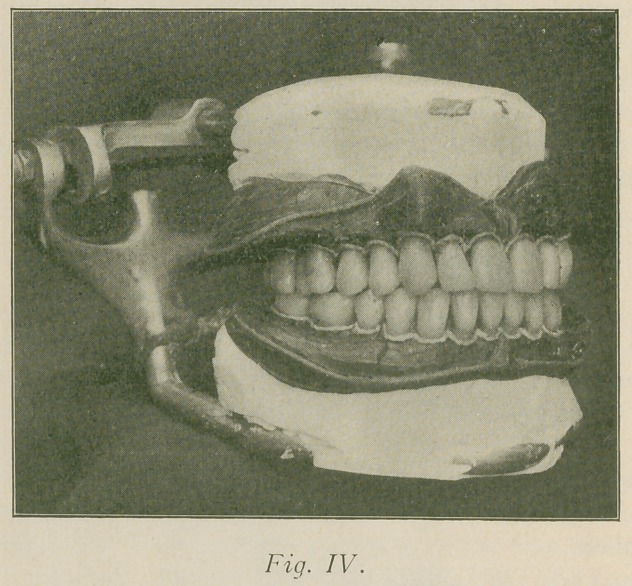


**Fig. V. f5:**
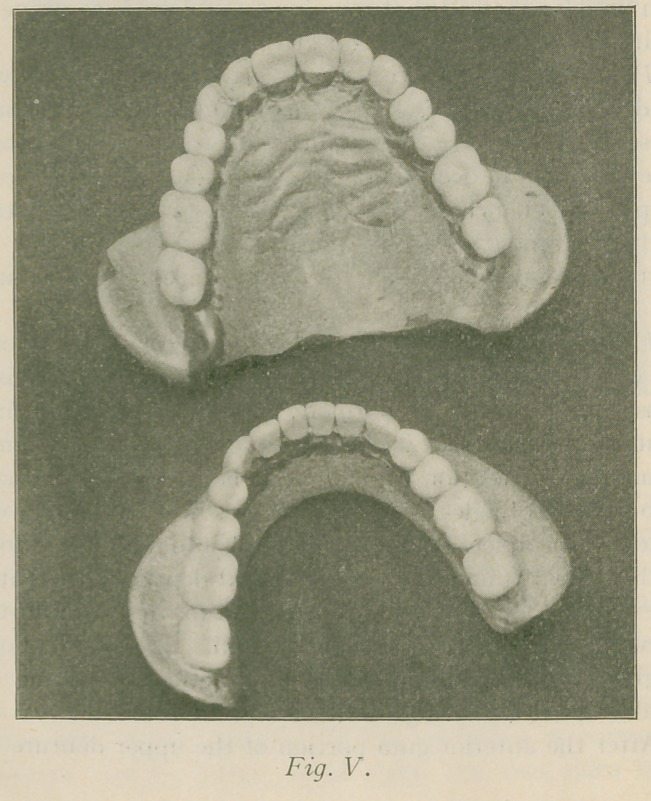


**Fig. VI. f6:**